# Validating emergency department cardioversion procedures in provincial administrative data in Ontario, Canada

**DOI:** 10.1371/journal.pone.0277598

**Published:** 2022-12-01

**Authors:** Clare L. Atzema, Ian G. Stiell, Alice S. Chong, Peter C. Austin

**Affiliations:** 1 Sunnybrook Health Sciences Centre, Toronto, ON, Canada; 2 ICES, Toronto, ON, Canada; 3 Division of Emergency Medicine, Department of Medicine, Sunnybrook Health Sciences Centre, Toronto, ON, Canada; 4 Institute for Health Policy, Management and Evaluation, Toronto, ON, Canada; 5 University of Toronto, Toronto, ON, Canada; 6 Ottawa University of Health Sciences, Ottawa, ON, Canada; Ohio State University, UNITED STATES

## Abstract

**Background:**

Cardioversion of acute-onset atrial fibrillation (AF) via electrical or pharmacological means is a common procedure performed in many emergency departments. While these procedures appear to be very safe, the rarity of subsequent adverse outcomes such as stroke would require huge sample sizes to confirm that conclusion. Big data can supply such sample sizes.

**Objective:**

We aimed to validate several potential codes for successful emergency department cardioversion of AF patients.

**Methods:**

This study combined 3 observational datasets of emergency department AF visits seen at one of 26 hospitals in Ontario, Canada, between 2008 and 2012. We linked patients who were eligible for emergency department cardioversion to several province-wide health administrative datasets to search for the associated cardioversion billing and procedural codes. Using the observational data as the gold standard for successful cardioversion, we calculated the test characteristics of a billing code (Z437) and of procedural codes 1.HZ.09JAFS and 1.HZ.09JAJS. Both include pharmacological and electrical cardioversions, as well as unsuccessful attempts; the latter is <10% using electricity (in Canada, standard practice is to proceed to electrical cardioversion if pharmacological cardioversion is unsuccessful).

**Results:**

Of 4557 unique patients in the three datasets, 2055 (45.1%) were eligible for cardioversion. Nine hundred thirty-three (45.4%) of these were successfully cardioverted to normal sinus rhythm. The billing code had slightly better test characteristics overall than the procedural codes. Positive predictive value (PPV) of a billing was 89.8% (95% CI, 87.0–92.2), negative predictive value (NPV) 70.5% (95% CI, 68.1–72.8), sensitivity 52.1% (95% CI, 48.8–55.3), and specificity 95.1% (95% CI, 93.7–96.3).

**Conclusions:**

AF patients who have been successfully cardioverted in an emergency department can be identified with high PPV and specificity using a billing code. Studies that require high sensitivity for cardioversion should consider other methods to identify cardioverted patients.

## Introduction

Over 33 million persons have atrial fibrillation (AF) worldwide [1], and many of these will present to an emergency department when they experience some of the potentially serious symptoms associated with AF, such as palpitations, chest tightness, and shortness of breath [2,3]. Guidelines recommend cardioversion as an management option for most acute-onset non-valvular AF (NVAF) patients, and in particular for new-onset AF and younger, highly symptomatic individuals [4–6].

Following publication of a retrospective study that suggested an increased risk of stroke and systemic thromboembolism (SSE) in non-anticoagulated patients cardioverted after AF duration > 12 hours [7], there has been controversy around elective cardioversion in non-anticoagulated patients with AF duration < 48 hours [8,9]. With only 38 events following > 5000 cardioversions, few confounders could be included in the model. Thus guideline recommendations around the timing of cardioversion of acute-onset NVAF are based on weak evidence [4].

Unfortunately, an estimated sample size of ~60,000 is needed to adequately power a study on the true risk of 30-day SSE in cardioverted emergency department NVAF patients; it is likely that only big data could the supply this sample size. Without validation of codes, however, erroneous conclusions can be drawn from analyses using administrative data. We aimed to assess the validity of several codes to identify emergency department AF cardioversion using administrative data.

## Methods

### Study design

This retrospective cohort study combined three existing clinical datasets of patients with a primary emergency department diagnosis of AF who were seen at one of 26 hospitals in Ontario, Canada, between 2008 and 2012. These 26 hospitals included a representative sample of academic, community, and small sites from Ontario. Patients were linked to province-wide administrative health datasets held at ICES using unique, encoded identifiers. Research Ethics Board approval was obtained from each of the sites; the need for patient consent was waived by all sites.

### Data sources and patient selection

Two datasets were collected from The Ottawa Hospital: both were chart reviews of patients eligible for acute cardioversion. One was conducted at the Ottawa Civic site in 2008 and the other from both the Ottawa Hospital’s Civic and General sites between 2010–2012 [3,10]. Inclusion criteria were all patients who were eligible for emergency department cardioversion, regardless of whether they received it; eligibility was defined as either duration NVAF (or atrial flutter) < 48 hours, or if it was < 7 days they were appropriately anticoagulated, or if not then a transthoracic echocardiogram could be obtained to rule out an atrial clot. Patients could be entered more than once; however, to be consistent with the third dataset, repeat visits were excluded and only the first visit made by an individual patient was retained for the current study.

The third dataset was created by identifying unique patients in the Canadian Institute for Health Information’s National Ambulatory Care Reporting System (NACRS) with a primary emergency department diagnosis of atrial fibrillation (ICD-10 code I48) between April 1/08 and March 30/09 at one of 24 Ontario hospitals. Sites were selected in a way that ensured a representative sample of hospital types (see S1 File). NACRS collects over 300 datapoints on the vast majority of all emergency department visits made in Ontario (data collection is mandatory for all Ontario hospitals). A trained abstractor went to each of the 24 hospitals and abstracted the charts of the identified AF patients. In all datasets electrical and pharmacological cardioversion attempts were recorded, along with success rates, and discharge status, amongst other variables.

### Data analysis

The three clinical datasets were linked to the Ontario Health Insurance Plan (OHIP) to search for a cardioversion billing code (Z437) and to NACRS for Canadian Classification of Health Interventions (CCI) procedural codes 1.HZ.09JAFS and 1.HZ.09JAJS. In Ontario, medically necessary care for Ontario residents is paid for by the provincial government; private funding is prohibited. In emergency departments, all physicians who work at the same hospital site will either bill entirely “fee-for-service” (i.e. bill OHIP for every patient visit and procedure, including the date of billing), or they will bill only a percentage of the procedure (e.g. 35%) in addition to being remunerated with an hourly rate. The latter approach is typically adopted in academic hospitals, which have teaching commitments, as it compensates physicians for the time spent teaching that could have been used to see (and bill for) more patients. Prior to 2008, physicians at academic sites in the province did not get paid at all via fee-for-service codes, although they were asked to ‘shadow-bill’ in order to make a record of what was done; however, since there was no monetary incentive to spend the time to shadow-bill, it was often done poorly and was not accurate at academic/teaching sites. In 2008 this was changed to incentivize physicians at these sites to see more patients, by allowing them to bill 25% of a fee-for-service billing code, and in 2009 it became 35%, where it remains today.

The process of learning how to bill at these sites has likely been gradual. While all cardioversion attempts (whether successful or not) can be billed to OHIP, anecdotally some emergency physicians were unaware that they could bill for an electrical cardioversion attempt that did not restore sinus rhythm (<10% of attempts), and also for pharmacological cardioversions (both successful and unsuccessful). If a pharmacological cardioversion is unsuccessful (~40% of attempts), in Canada it is very likely that the managing physician will proceed to electrical cardioversion, as per the Aggressive Ottawa Protocol [11,12] (which is >90% successful, and potentially higher because it follows administration of antiarrhythmic medications [13,14]). Thus the Z437 billing code represents attempted cardioversions, while our gold standard was successful cardioversion; therefore, the billing code will have slightly reduced positive predictive value (PPV) and specificity due to false positives billed by the managing physician when in fact the cardioversion was not successful.

In comparison, lack of billing for a successful cardioversion (either pharmacological or electrical) will result in false negatives, and reduced sensitivity and negative predictive value (NPV) for detection of successful cardioversion. However, lack of billing due to unawareness that an unsuccessful cardioversion can still be billed to OHIP would *not* represent a false negative, as the gold standard is *successful* cardioversion.

Procedural codes are not subject to the issues around billing of unsuccessful procedures, but are limited by the accuracy of the NACRS abstractor at each hospital to capture cardioversion attempts from the emergency department charts. All NACRS abstractors undergo formal standardized training in how to code, including how to code for missing chart data.

To allow for cardioversions that occurred close to midnight, we included codes that were dated +/- 1 day of emergency department arrival. Descriptive statistics were used to describe the study cohort by cardioversion status. Test characteristics, including PPV, NPV, sensitivity, and specificity of both the billing code, and of the procedural codes, were calculated along with 95% confidence intervals (CI). To assess whether the sensitivity using the billing code Z437 was different in the several years that followed the introduction of OHIP billing at academic sites in 2008, we performed a χ^2^ test on the test characteristics of the cohort seen annually between 2008 and 2012. Similarly, to assess for heterogeneity of the test characteristics for the billing code Z437 by hospital site, we used a χ^2^ test. The same analyses were repeated for the procedural codes. Analyses were performed with SAS software (Version 9.3, SAS Institute Inc., Cary, NC).

## Results

There were 4557 unique emergency department NVAF patients seen at the 26 hospitals between 2008 and 2012 and included in the three clinical datasets, of whom 2055 (45.1%) were eligible for cardioversion. Median age was 66.0 years and 47.9% were female (Table 1). Nine hundred thirty-three (45.4%) were successfully cardioverted to normal sinus rhythm, including 351 (37.6%) pharmacological and 582 (62.4%) electrical cardioversions.

**Table 1:** Descriptive characteristics of patients by receipt of emergency department cardioversion.

		All (%)	Cardioverted (%)	Not cardioverted (%)	
Characteristic		n = 2055	n = 933 (45.4)	n = 1122 (54.6)	p
**Demographics**					
Age	Mean ± SD	64.48 ± 14.97	60.76 ± 14.82	67.58 ± 14.38	<0.001
	Median (IQR)	66 (55–76)	61 (51–72)	69 (59–79)	<0.001
Female sex		985 (47.9)	376 (40.3)	609 (54.3)	<0.001
**Past Medical History**					
Atrial fibrillation		1,283 (62.4)	588 (63.0)	695 (61.9)	<0.001
Heart failure		77 (3.7)	14 (1.5)	63 (5.6)	<0.001
Hypertension		947 (46.1)	367 (39.3)	580 (51.7)	<0.001
Diabetes Mellitus		252 (12.3)	83 (8.9)	169 (15.1)	<0.001
Stroke or TIA		106 (5.2)	25 (2.7)	81 (7.2)	<0.001
Coronary artery disease		316 (15.4)	127 (13.6)	189 (16.8)	0.04
Valvular disease		147 (7.2)	50 (5.4)	97 (8.6)	0.004
Chronic Obstructive Pulmonary Disease		98 (4.8)	43 (4.6)	55 (4.9)	0.76
Chronic renal failure		46 (2.2)	20 (2.1)	26 (2.3)	0.79
CHADS2 score	2+	592 (28.8)	172 (18.4)	420 (37.4)	<0.001
CHA_2_DS_2_-VASc score	0	414 (20.1)	258 (20.7)	156 (13.9)	<0.001
	1	438 (21.3)	238 (25.5)	200 (17.8)	< .001
	2	361 (17.6)	168 (18.0)	193 (17.2)	0.633
	3	360 (17.5)	130 (13.9)	230 (20.5)	< .001
	4+	482 (23.5)	139 (14.9)	343 (30.6)	< .001
HAS-BLED score 4+		42 (2.0)	12 (1.3)	30 (2.7)	0.03
**Emergency department**					
Arrival by ambulance		531 (25.8)	209 (22.4)	322 (28.7)	0.003
Triage Score^a^	1/2	1,704 (82.9)	804 (86.2)	900 (80.2)	<0.001
Presenting heart rate*^28 missing^	Median (IQR)	118 (91–140)	120 (92–140)	117 (90–140)	0.09
Presenting systolic BP*^40 missing^	Median (IQR)	133 (119–149)	131 (117–147)	135 (120–151)	<0.001
Creatinine (umol/L)	Median (IQR)	82 (69–97)	81 (68–94)	82 (70–99)	0.01

SD: Standard deviation; IQR: Interquartile range; TIA: Transient Ischemic Attack.

^a^ Using the Canadian Triage and Acuity Score (CTAS) [15].

* Number of missing datapoints for that variable.

The billing code had slightly better test characteristics overall than the procedural codes (Table 2). For the Z437 billing code: PPV 90%; specificity 95%; NPV 71%; sensitivity 52%. In comparison, the procedure codes yielded a similar PPV and specificity of 92% and of 97%, respectively, with an NPV 66% but a 12% lower sensitivity at 40%.

**Table 2:** Test characteristics of cardioversion codes.

**Billing code Z437**						
	**Successful CV**	**No CV (or CV unsuccessful)**		**Test Characteristics**	**%**	**95% CI**
Billing of Z437	486	55	541	PPV	89.8	87.0–92.2
No billing	447	1067	1514	NPV	70.5	68.1–72.8
Total	933	1122	2055	Sensitivity	52.1	48.8–55.3
				Specificity	95.1	93.7–96.3
**CCI codes 1.HZ.09JAFS and 1.HZ.09JAJS**						
	**Successful CV**	**No CV (or CV unsuccessful)**		**Test Characteristics**	**%**	**95% CI**
+ CCI codes	373	32	405	PPV	92.1	89.0–94.5
No CCI codes	560	1090	1650	NPV	66.1	63.7–68.3
Total	933	1122	2055	Sensitivity	40.0	36.8–43.2
				Specificity	97.1	96.0–98.0

CV: Cardioversion; CI: Confidence interval; PPV: Positive predictive value; NPV: Negative predictive value; CCI: Canadian Classification of Health Interventions.

The sensitivity of the billing code changed significantly between 2008 and 2012, improving from 45% to 74% (Table 3). Specificity and NPV decreased somewhat, likely due to increased use of cardioversion in AF patients over time (which would decrease the number of true negatives). PPV did not change significantly. Using procedure codes, the results were similar for PPV, NPV, and specificity, while sensitivity of procedure codes did not change significantly over time.

**Table 3:** Test characteristics of cardioversion billing code Z437 and CCI codes 1.HZ.09JAFS, 1.HZ.09JAJS, over the study period.

	Sensitivity	p	Specificity	p	PPV	p	NPV	p
**Billing code Z437**								
2008	44.9%	<0.001	96.4%	<0.001	88.6%	0.89	73.8%	<0.001
2009	49.2%		97.4%		90.9%		78.5%	
2010	56.4%		85.3%		91.4%		41.1%	
2011	65.1%		78.1%		90.1%		42.4%	
2012	74.4%		86.7%		93.5%		56.5%	
**CCI codes 1.HZ.09JAFS & 1.HZ.09JAJS**								
2008	38.9%	0.97	98.2%	<0.001	93.1%	0.36	72.1%	<0.001
2009	41.0%		98.3%		92.6%		76.0%	
2010	40.4%		91.2%		92.7%		35.6%	
2011	41.5%		89.1%		92.0%		33.3%	
2012	41.0%		73.3%		80.0%		32.4%	

PPV: Positive predictive value; NPV: Negative predictive value; CCI: Canadian Classification of Health Interventions.

There was variation in test characteristics across the 26 academic, community, and small hospital emergency departments (p<0.001), with the exception of PPV when using the procedure codes (p = 0.22) (See S2 File).

## Discussion

Dramatic increases in the prevalence of AF are predicted in the upcoming decades [16,17], which will result in many more patients undergoing emergency department cardioversion in the future. Population-level assessments of the real-world safety of this procedure are critical to optimize patient care; however, without validation of the codes used in large datasets there is potential for gross misclassification, which could in turn lead to false study conclusions. In this study we confirmed that billing codes can identify cardioverted emergency department AF patients with high PPV and specificity in big data. We found that procedural codes were slightly less accurate than billing codes.

Study of emergency department cardioversion has been limited by low event rates of the major adverse events of concern, stroke and systemic thromboembolism [4]. Our study demonstrates that large numbers of cardioverted emergency department AF patients can be identified with high PPV and specificity in big data, facilitating the sample size required to establish subsequent stroke rates in future studies.

While the PPV and specificity of the billing code for cardioversion was high, almost half of patients who were cardioverted were not identified using the billing code (sensitivity 52%). The NPV was 71%, meaning that 29% of the cohort who were classified using the code as *not* having been cardioverted actually were successfully cardioverted (likely due to unbilled cardioversions). Thus, direct comparison of patients who the code identifies as having undergone successful cardioversion to those whom the code specifies have not will be limited by misclassification bias, and bias toward the null. This will be less of an issue in regions in which physicians are well-remunerated for performing cardioversion and/or are aware that pharmacological cardioversion is an appropriate procedural billing, which was likely not the case in Ontario the early part of our study period. Indeed, we found that the billing code sensitivity increased by almost 30% even by 2012, four years after fee-for-service billing was introduced at academic sites (which are the sites where cardioversion is most likely to occur) [18]. By comparison, sensitivity for procedural codes, for which data collection did not change over time, was not different. We surmise that if the study were repeated now in Ontario, where remuneration via billings for cardioversion has been in place for over a decade and the fee is high relative to most billings, sensitivity would be substantially higher.

Inpatient AF procedures have also been validated in large datasets, and test characteristics are high, with PPV of ablation at 98.8% and sensitivity of 87.2%, while a diagnostic electrophysiology study (EPS) had a lower PPV at 88.0% and sensitivity of 61.1% [19]. The diagnosis of AF itself has been validated in large databases including NACRS, with good PPV and sensitivity [20]; its high PPV is likely secondary to the fact that there is a simple test to identify the AF that is available at the time of the emergency department visit (i.e. an electrocardiogram), unlike other diagnoses such as a transient ischemic attack [21].

Our study has limitations. The data in this study are over a decade old; however, the billing and procedural codes for cardioversion have not changed in that time. Regions that remunerate physicians without a fee-for-service component are likely to have worse sensitivity using the billing code (which would be entirely reliant on shadow-billing, in that case); procedural codes may be preferential in those areas, although accuracy of administrative data will vary by dataset, and we only assessed Ontario data (in NACRS) in our study. Our billings data relied on billings from an entire province with universal healthcare coverage; our findings may not apply to datasets without this coverage, nor to datasets of private insurance billings, which may have variable billing incentives and/or quality checks. Studies like this one are needed to validate the billings in other types of datasets, and need to be updated over time when billing procedures change. We did not assess whether the cardioversion was billed by an emergency physician or a cardiologist.

## Conclusions

Successful cardioversions performed in emergency departments can be identified with high specificity and PPV using administrative data.

## Supporting information

S1 File Emergency departments included in the study cohort. (DOCX)

S2 File Heterogeneity of test characteristics by site. (DOCX)
