# Supplementary material for: Validating emergency department cardioversion procedures in provincial administrative data in Ontario, Canada
Source: PLoS One. 2022 Dec 1;17(12):e0277598. doi: 10.1371/journal.pone.0277598 (PMC9714737; doi:10.1371/journal.pone.0277598)
Supplement: S1 File — (DOCX) [file pone.0277598.s001.docx]

**S1.** **Emergency departments and sample size by site in the eligible study cohort**

|  | Hospital Name | n |
| --- | --- | --- |
| 1 | St Michael's Hospital | 59 (2.9) |
| 2 | Credit Valley Hospital | 99 (4.8) |
| 3 | Southlake Regional Health Centre | 149 (7.3) |
| 4 | Guelph General Hospital | 48 (2.3) |
| 5 | Ottawa Hospital - Civic Site | 307 (14.9) |
| 6 | Ottawa Hospital - General Site | 172 (8.4) |
| 7 | Humber River Regional Hospital - York-Finch | 32 (1.6) |
| 8 | Trillium Health Centre - Mississauga | 100 (4.9) |
| 9 | Cambridge Memorial Hospital | 54 (2.6) |
| 10 | Mackenzie Health - Richmond Hill Hospital | 98 (4.8) |
| 11 | Lakeridge Health - Oshawa Site | 125 (6.1) |
| 12 | Lakeridge Health - Port Perry | 14 (0.7) |
| 13 | Halton Healthcare Services Corporation - Milton | 18 (0.9) |
| 14 | Sunnybrook Health Sciences Centre | 136 (6.6) |
| 15 | Hamilton Health Sciences Corporation - General | 68 (3.3) |
| 16 | North York General Hospital | 133 (6.5) |
| 17 | North York General Hospital-Branson Site | 7 (0.3) |
| 18 | Markham Stouffville Hospital | 75 (3.6) |
| 19 | William Osler Health System - Etobicoke | 47 (2.3) |
| 20 | Stevenson Memorial Hospital Alliston | 18 (0.9) |
| 21 | University Health Network | 80 (3.9) |
| 22 | University Health Network - Western Site | 56 (2.7) |
| 23 | Trillium Health Centre - West Toronto | 19 (0.9) |
| 24 | Markham Stouffville Hospital - Uxbridge Site | 13 (0.6) |
| 25 | Brant Community Healthcare System - Brantford | 53 (2.6) |
| 26 | William Osler Health System - Civic Site | 75 (3.6) |
|  | Total | 2055 |

Hospital Selection: For the third dataset, created from NACRS (24 sites, which did not include The Ottawa Hospitals), in order to create a sample that allowed us to generalize our results to all Ontario AF patients, we used a two-stage stratified sampling strategy. First, we categorized sites into four different strata: tertiary sites, large community sites (> 200 AF visits/year), intermediate community (101 to 200 AF visits/year), and small sites (≤ 100/year). Due to study funding constraints, we had to limit our sites to those that were within daily driving distance (~80 miles) of the Greater Toronto Area, or that had a sister site that could be accessed within that radius. We excluded specialized emergency departments (e.g. solely pediatric or psychiatric) and very small sites (<25 AF visits/year), as the latter are not usually open 24 hours/day and see a much lower acuity patient. At the first sampling stage, we selected a random sample of sites from each stratum. At the second stage, using the CIHI-NACRS list of eligible patients at each site, we abstracted a random sample of charts for abstraction from each of the selected hospital sites.(2)

**S2. Heterogeneity of test characteristics by site**

| **By procedure codes 1.HZ.09JAFS and 1.HZ.09JAJS** | | | |  |  |  |  |  |  |  |  |  |  |  |  |  |
| --- | --- | --- | --- | --- | --- | --- | --- | --- | --- | --- | --- | --- | --- | --- | --- | --- |
| **Hospital emergency department site** | **PPV (%)** | **95 CI** | | **p** | **NPV (%)** | **95 CI** | | **p** | **Sens (%)** | **95 CI** | | **p** | **Spec (%)** | **95 CI** | | **p** |
| St Michael's Hospital | 100.0 | 15.8 | 100.0 | 0.216 | 68.4 | 54.8 | 80.1 | <.001 | 10.0 | 1.2 | 31.7 | <.001 | 100.0 | 91.0 | 100.0 | <.001 |
| Credit Valley Hospital | 71.4 | 29.0 | 96.3 | 0.216 | 63.0 | 52.3 | 72.9 | <.001 | 12.8 | 4.3 | 27.4 | <.001 | 96.7 | 88.5 | 99.6 | <.001 |
| Southlake Regional Health Centre | 97.1 | 89.9 | 99.6 | 0.216 | 70.0 | 58.7 | 79.7 | <.001 | 73.6 | 63.3 | 82.3 | <.001 | 96.6 | 88.1 | 99.6 | <.001 |
| Guelph General Hospital | 100.0 | 59.0 | 100.0 | 0.216 | 82.9 | 67.9 | 92.8 | <.001 | 50.0 | 23.0 | 77.0 | <.001 | 100.0 | 89.7 | 100.0 | <.001 |
| Ottawa Hospital - Civic & General site Site | 90.6 | 85.2 | 94.5 | 0.216 | 35.0 | 29.6 | 40.6 | <.001 | 43.4 | 38.2 | 48.7 | <.001 | 87.1 | 79.9 | 92.4 | <.001 |
| Humber River Regional Hospital -York-Finch | 100.0 | 15.8 | 100.0 | 0.216 | 80.0 | 61.4 | 92.3 | <.001 | 25.0 | 3.2 | 65.1 | <.001 | 100.0 | 85.8 | 100.0 | <.001 |
| Trillium Health Centre - Mississauga | 90.9 | 58.7 | 99.8 | 0.216 | 68.5 | 57.8 | 78.0 | <.001 | 26.3 | 13.4 | 43.1 | <.001 | 98.4 | 91.3 | 100.0 | <.001 |
| Cambridge Memorial Hospital | 80.0 | 28.4 | 99.5 | 0.216 | 87.8 | 75.2 | 95.4 | <.001 | 40.0 | 12.2 | 73.8 | <.001 | 97.7 | 88.0 | 99.9 | <.001 |
| Mackenzie Health - Richmond Hill Hospital | 100.0 | 82.4 | 100.0 | 0.216 | 65.8 | 54.3 | 76.1 | <.001 | 41.3 | 27.0 | 56.8 | <.001 | 100.0 | 93.2 | 100.0 | <.001 |
| Lakeridge Health - Oshawa Site | 84.6 | 69.5 | 94.1 | 0.216 | 81.4 | 71.6 | 89.0 | <.001 | 67.3 | 52.5 | 80.1 | <.001 | 92.1 | 83.6 | 97.0 | <.001 |
| Lakeridge Health - Port Perry | * | * | * | . | * | * | * | <.001 | * | * | * | <.001 | * | * | * | <.001 |
| Halton Healthcare Services Corporation - Milton | * | * | * | 0.216 | * | * | * | <.001 | * | * | * | <.001 | * | * | * | <.001 |
| Sunnybrook Health Sciences Centre | 100.0 | 75.3 | 100.0 | 0.216 | 72.4 | 63.6 | 80.0 | <.001 | 27.7 | 15.6 | 42.6 | <.001 | 100.0 | 95.9 | 100.0 | <.001 |
| Hamilton Health Sciences Corporation - General | 100.0 | 15.8 | 100.0 | 0.216 | 83.3 | 72.1 | 91.4 | <.001 | 15.4 | 1.9 | 45.4 | <.001 | 100.0 | 93.5 | 100.0 | <.001 |
| North York General Hospital | 94.7 | 74.0 | 99.9 | 0.216 | 64.0 | 54.5 | 72.8 | <.001 | 30.5 | 19.2 | 43.9 | <.001 | 98.6 | 92.7 | 100.0 | <.001 |
| North York General Hospital-Branson Site | * | * | * | 0.216 | * | * | * | <.001 | * | * | * | <.001 | * | * | * | <.001 |
| Markham Stouffville Hospital | 100.0 | 78.2 | 100.0 | 0.216 | 76.7 | 64.0 | 86.6 | <.001 | 51.7 | 32.5 | 70.6 | <.001 | 100.0 | 92.3 | 100.0 | <.001 |
| William Osler Health System - Etobicoke | 66.7 | 22.3 | 95.7 | 0.216 | 85.4 | 70.8 | 94.4 | <.001 | 40.0 | 12.2 | 73.8 | <.001 | 94.6 | 81.8 | 99.3 | <.001 |
| Stevenson Memorial Hospital Alliston | * | * | * | . | * | * | * | <.001 | * | * | * | <.001 | * | * | * | <.001 |
| University Health Network | 100.0 | 29.2 | 100.0 | 0.216 | 62.3 | 50.6 | 73.1 | <.001 | 9.4 | 2.0 | 25.0 | <.001 | 100.0 | 92.6 | 100.0 | <.001 |
| University Health Network - Western Site | 66.7 | 9.4 | 99.2 | 0.216 | 75.5 | 61.7 | 86.2 | <.001 | 13.3 | 1.7 | 40.5 | <.001 | 97.6 | 87.1 | 99.9 | <.001 |
| Trillium Health Centre - West Toronto | * | * | * | . | * | * | * | <.001 | * | * | * | <.001 | * | * | * | <.001 |
| Markham Stouffville Hospital - Uxbridge Site | * | * | * | 0.216 | * | * | * | <.001 | * | * | * | <.001 | * | * | * | <.001 |
| Brant Community Healthcare System - Brantford | 100.0 | 2.5 | 100.0 | 0.216 | 73.1 | 59.0 | 84.4 | <.001 | 6.7 | 0.2 | 31.9 | <.001 | 100.0 | 90.7 | 100.0 | <.001 |
| William Osler Health System - Civic Site | 100.0 | 63.1 | 100.0 |  | 73.1 | 60.9 | 83.2 |  | 30.8 | 14.3 | 51.8 |  | 100.0 | 92.7 | 100.0 |  |

| **By Billing code Z437** |  |  |  |  |  |  |  |  |  |  |  |  |  |  |  |  |
| --- | --- | --- | --- | --- | --- | --- | --- | --- | --- | --- | --- | --- | --- | --- | --- | --- |
| **Hospital emergency department site** | **PPV (%)** | **95 CI** | | **p** | **NPV (%)** | **95 CI** | | **p** | **Sens (%)** | **95 CI** | | **p** | **Spec (%)** | **95 CI** | | **p** |
| St Michael's Hospital | 75.0 | 19.4 | 99.4 | <.001 | 69.1 | 55.2 | 80.9 | <.001 | 15.0 | 3.2 | 37.9 | <.001 | 97.4 | 86.5 | 99.9 | <.001 |
| Credit Valley Hospital | 91.7 | 61.5 | 99.8 | <.001 | 67.8 | 56.9 | 77.4 | <.001 | 28.2 | 15.0 | 44.9 | <.001 | 98.3 | 91.1 | 100.0 | <.001 |
| Southlake Regional Health Centre | 98.5 | 91.8 | 100.0 | <.001 | 68.7 | 57.6 | 78.4 | <.001 | 71.4 | 61.0 | 80.4 | <.001 | 98.3 | 90.8 | 100.0 | <.001 |
| Guelph General Hospital | 100.0 | 47.8 | 100.0 | <.001 | 79.1 | 64.0 | 90.0 | <.001 | 35.7 | 12.8 | 64.9 | <.001 | 100.0 | 89.7 | 100.0 | <.001 |
| Ottawa Hospital - Civic & General site Site | 91.1 | 86.9 | 94.4 | <.001 | 44.2 | 37.6 | 50.8 | <.001 | 63.7 | 58.4 | 68.7 | <.001 | 82.3 | 74.4 | 88.5 | <.001 |
| Humber River Regional Hospital -York-Finch | 100.0 | 47.8 | 100.0 | <.001 | 88.9 | 70.8 | 97.6 | <.001 | 62.5 | 24.5 | 91.5 | <.001 | 100.0 | 85.8 | 100.0 | <.001 |
| Trillium Health Centre - Mississauga | 70.0 | 45.7 | 88.1 | <.001 | 70.0 | 58.7 | 79.7 | <.001 | 36.8 | 21.8 | 54.0 | <.001 | 90.3 | 80.1 | 96.4 | <.001 |
| Cambridge Memorial Hospital | 50.0 | 1.3 | 98.7 | <.001 | 82.7 | 69.7 | 91.8 | <.001 | 10.0 | 0.3 | 44.5 | <.001 | 97.7 | 88.0 | 99.9 | <.001 |
| Mackenzie Health - Richmond Hill Hospital | 100.0 | 84.6 | 100.0 | <.001 | 68.4 | 56.7 | 78.6 | <.001 | 47.8 | 32.9 | 63.1 | <.001 | 100.0 | 93.2 | 100.0 | <.001 |
| Lakeridge Health - Oshawa Site | 78.8 | 61.1 | 91.0 | <.001 | 75.0 | 64.9 | 83.4 | <.001 | 53.1 | 38.3 | 67.5 | <.001 | 90.8 | 81.9 | 96.2 | <.001 |
| Lakeridge Health - Port Perry | * | * | * | . | * | * | * | <.001 | * | * | * | <.001 | * | * | * | <.001 |
| Halton Healthcare Services Corporation - Milton | * | * | * | . | * | * | * | <.001 | * | * | * | <.001 | * | * | * | <.001 |
| Sunnybrook Health Sciences Centre | 100.0 | 79.4 | 100.0 | <.001 | 74.2 | 65.4 | 81.7 | <.001 | 34.0 | 20.9 | 49.3 | <.001 | 100.0 | 95.9 | 100.0 | <.001 |
| Hamilton Health Sciences Corporation - General | 50.0 | 1.3 | 98.7 | <.001 | 81.8 | 70.4 | 90.2 | <.001 | 7.7 | 0.2 | 36.0 | <.001 | 98.2 | 90.3 | 100.0 | <.001 |
| North York General Hospital | 87.2 | 72.6 | 95.7 | <.001 | 73.4 | 63.3 | 82.0 | <.001 | 57.6 | 44.1 | 70.4 | <.001 | 93.2 | 84.9 | 97.8 | <.001 |
| North York General Hospital-Branson Site | * | * | * | . | * | * | * | <.001 | * | * | * | <.001 | * | * | * | <.001 |
| Markham Stouffville Hospital | 100.0 | 76.8 | 100.0 | <.001 | 75.4 | 62.7 | 85.5 | <.001 | 48.3 | 29.4 | 67.5 | <.001 | 100.0 | 92.3 | 100.0 | <.001 |
| William Osler Health System - Etobicoke | 66.7 | 9.4 | 99.2 | <.001 | 81.8 | 67.3 | 91.8 | <.001 | 20.0 | 2.5 | 55.6 | <.001 | 97.3 | 85.8 | 99.9 | <.001 |
| Stevenson Memorial Hospital Alliston | * | * | * | . | * | * | * | <.001 | * | * | * | <.001 | * | * | * | <.001 |
| University Health Network | 100.0 | 76.8 | 100.0 | <.001 | 72.7 | 60.4 | 83.0 | <.001 | 43.8 | 26.4 | 62.3 | <.001 | 100.0 | 92.6 | 100.0 | <.001 |
| University Health Network - Western Site | 83.3 | 35.9 | 99.6 | <.001 | 80.0 | 66.3 | 90.0 | <.001 | 33.3 | 11.8 | 61.6 | <.001 | 97.6 | 87.1 | 99.9 | <.001 |
| Trillium Health Centre - West Toronto | * | * | * | . | * | * | * | <.001 | * | * | * | <.001 | * | * | * | <.001 |
| Markham Stouffville Hospital - Uxbridge Site | * | * | * | 0.216 | * | * | * | <.001 | * | * | * | <.001 | * | * | * | <.001 |
| Brant Community Healthcare System - Brantford | 0.0 | 0.0 | 97.5 | <.001 | 71.2 | 56.9 | 82.9 | <.001 | 0.0 | 0.0 | 21.8 | <.001 | 97.4 | 86.2 | 99.9 | <.001 |
| William Osler Health System - Civic Site | 94.1 | 71.3 | 99.9 | <.001 | 82.8 | 70.6 | 91.4 | <.001 | 61.5 | 40.6 | 79.8 | <.001 | 98.0 | 89.1 | 99.9 | <.001 |

PPV: positive predictive value; CI: confidence interval; NPV: negative predictive value; Sens: sensitivity; Spec: specificity

* small cell size: not reported due to agreement between ICES and the Canadian Institutes of Health Research, for privacy reasons
